# Individual and environmental correlates of objectively measured physical activity and sedentary time in adults from Curitiba, Brazil

**DOI:** 10.1007/s00038-017-0995-0

**Published:** 2017-07-17

**Authors:** Priscila Bezerra Gonçalves, Pedro Curi Hallal, Adriano Akira Ferreira Hino, Rodrigo Siqueira Reis

**Affiliations:** 1 Physical Activity and Quality of Life Research Group (GPAQ), Pontifical Catholic University of Paraná, Curitiba, PR Brazil; 2 Health Technology Graduate Program, Pontifical Catholic University of Paraná, Curitiba, PR Brazil; 3 Post-Graduate Program in Epidemiology, Federal University of Pelotas, Pelotas, RS Brazil; 4 Prevention Research Center, Brown School, Washington University in St. Louis, St. Louis, MO USA

**Keywords:** Individual correlates, Environmental correlates, Adults, Accelerometry, Physical activity, Sedentary time

## Abstract

**Objectives:**

This study assessed the association of individual and neighborhood environment characteristics and objectively measured physical activity (PA) and sedentary time (ST) in adults from Curitiba, Brazil.

**Methods:**

A population-based cross-sectional study was conducted through face-to-face household interviews in 2010. The analytic sample included 305 adults aged 20–65 years recruited from 32 census tracts selected according to neighborhood walkability and socioeconomic status. Individual and environmental PA correlates were evaluated through standardized and valid self-reported measures, including the Neighborhood Environment Walkability Scale. Minutes per week of PA and ST were assessed through accelerometry. Multi-level regression models were used in the analyses.

**Results:**

After adjusting for confounders the strongest individual and environmental correlates associated with ST was residential density (*B* = 0.14; *p* = 0.008), light-intensity PA was being a father/mother (*B* = 35.71; *p* = 0.025) and moderate-to-vigorous PA was sex (*B* = 0.91; *p* < 0.001) and number of cars (one car, *B* = −1.05; two cars, *B* = −1.14; *p* < 0.001).

**Conclusions:**

The associations found with individual and environmental correlates varied accordingly across all outcomes. Future changes in policies and infrastructure should consider the social context of the community and improvements to promote a safer environment in the neighborhood.

## Introduction

Physical inactivity, defined as a condition of not reaching the public health guidelines for the recommended levels of moderate to vigorous physical activity (Hallal et al. 2012), has been identified as one of the most important threats to public health in contemporary societies (Kohl et al. 2012). Nearly one-fourth of the adults and four-fifths of the adolescents worldwide are physically inactive (Sallis et al. 2016) and 5.3 million deaths per year could be prevented through the promotion of physical activity (Hallal et al. 2012). Societal megatrends, including economic, social and technological changes have led to decreases in daily energy expenditure (CDC 1999). This situation is particularly concerning in rapid transitioning societies such as Brazil, in which non-communicable diseases have rapidly replaced infectious diseases as the main causes of death (Schmidt et al. 2012). In addition, in Brazil greater healthcare expenditures due to drug treatments was associated with lower levels of physical activity (Codogno et al. 2015).

A growing and consistent body of evidence indicates that sedentary behavior is a strong predictor of several health problems, including obesity and mortality, regardless of the physical activity level (Healy et al. 2011; Van Dyck et al. 2012). Sedentary behavior refers to any behavior performed in a sitting or reclining posture and up to 1.5 metabolic equivalents (METs) (Owen et al. 2014). More recently, sedentary behavior combined with low level of physical activity has been identified as a strong predictor of mortality rates (Ekelund et al. 2016). Furthermore, the literature suggests that some individual characteristics and environmental attributes are associated with sedentary behavior (Van Dyck et al. 2012; Koohsari et al. 2015).

Understanding the correlates and determinants of physical activity and sedentary behavior is essential to help increase the effectiveness and tailoring of interventions (Reis et al. 2016). However, to the date most studies investigating these correlates have relied on cross-sectional data of individual-level variables related to physical activity behaviors (Bauman et al. 2012). In addition, despite the increasing number of studies on environmental correlates of physical activity there is still scarce evidence emerging from developing countries (Hino et al. 2010; Sallis et al. 2016). Hence, there is limited evidence on how individual and environmental level characteristics related to physical activity and sedentary behavior may interact, particularly from low- and middle-income countries. Low- and middle- income countries have unique built and social environmental characteristics that could affect the associations with physical activity. For instance, Latin America is the most urbanized region in the world, with high densities (e.g. population, residential and streets) and land use mix combined with greater social inequalities and crime rates (Salvo et al. 2014a). Hence, the built environment in the region is somehow conducive to physical activity, whereas the social characteristics present major barriers to transport and leisure physical activity. The available evidence somehow supports the need for further examination on these associations. For instance, in high-income countries walkability is positively associated with leisure and transport physical activity (Van Dyck et al. 2010b, 2011), whereas in low- and middle- income countries walkability is inversely associated with physical activity (Salvo et al. 2014a, b). In addition, the effect sizes between walkability and physical activity vary substantially worldwide (Adams et al. 2014).

Consistent associations have been found between several features of the community environment (residential density, street connectivity, land use, safety and aesthetics) and physical activity (Bauman et al. 2012; Hino et al. 2010; Van Dyck et al. 2011). Additionally, access to physical activity facilities and equipment have been related to leisure time physical activity (Hino et al. 2011). However, the evidence on environment correlates of sedentary behavior is scarce and inconsistent. For instance, while individual characteristics (e.g. gender, age, education and marital status) have been positively associated with measures of physical activity and sedentary behavior (Van Dyck et al. 2012; 2013), some environmental attributes (e.g. residential density, access to services and aesthetics) have been found to be negatively associated with accelerometer measured sedentary time (Van Dyck et al. 2012). Furthermore, nearly all evidence on this field is emerging from high-income countries (Bauman et al. 2012).

Hence, a key literature gap in this area is the paucity of studies from low- and middle- income countries, particularly using objectively measured physical activity and the lack of studies that examining environmental correlates of sedentary behavior (Bauman et al. 2012; Van Dyck et al. 2010b). The use of objective measures is important since as it allows capturing time spent in physical activity and sedentary behaviors with more precision. In fact, self-reported measures overestimate both physical activity and sedentary time when compared to objective measures (Sallis et al. 2016). The aim of the present study was to evaluate the association between individual characteristics, features of the environment and objectively measured sedentary time and physical activity of adults living in the city of Curitiba, Brazil.

## Methods

### Sample and procedures

A population-based cross-sectional was conducted in the city of Curitiba, Brazil in 2010 through face-to-face household interviews. This survey was part of the International Physical Activity and Environment (IPEN) Study (Kerr et al. 2013). Individuals were selected through a multiple stages sampling approach. First, all census tracts (CT) were identified and classified using deciles of walkability and socioeconomic status (SES). Second, after cross comparisons four quadrants of walkability and SES were produced and eight CT within each quadrant were randomly selected (*N* = 32). Third, within each selected CT, eligible adults were randomly selected and a total of 699 participants completed the study. A subsample of 381 participants (54.4%) was selected to wear accelerometers (Hino et al. 2012). The compliance rate was 80.0% and losses (19.2%) were due to refusals (7.6%) and non-valid data after wearing the devices (11.6%). The final sample comprised 305 individuals aged 20–65 years living for at least one year in the same address. No selection bias has been found between the analytical and the initial samples. Further details about the methodology of the survey and the sampling strategy are available elsewhere (Hino et al. 2012).

### Environmental characteristics

The Brazilian version of the Neighborhood Environment Walkability Scale—Abbreviated or A-NEWS (Malavasi et al. 2007) was used to capture perception of environment. The final instrument comprised 85 questions including locally relevant questions and organized into nine community environment domains [residential density (presence of houses and apartments in neighborhood), land use mix (e.g. supermarket, restaurants, parks near at home), access to services (e.g. store are within easy walking distance to or from my home), streets connectivity (e.g. short distance between intersections in my neighborhood), walking and cycling facilities (e.g. presence of sidewalks), aesthetics (e.g. presence of trees, interesting things and attractive natural), neighborhood safety from pedestrian/traffic (much traffic along nearby streets), neighborhood safety from crime (crime rate) and general satisfaction with the neighborhood (e.g. like the neighborhood where living)]. The response scales include five (e.g. land use mix), two (e.g. general neighborhood satisfaction) and four points Likert-scales. Further description on the scale is available elsewhere (Cerin et al. 2013).

### Dependent variables

Physical activity and sedentary time were assessed through accelerometry. Two ActiGraph models were used (7164 and GT1M). A previous study (Kozey et al. 2010) showed that the use of the two models provides comparable activity counts. Additionally, accelerometry has already been shown to be valid to assess physical activity in Brazilian adults (Bennett et al. 2007; Reichert et al. 2009). Participants were instructed to wear the accelerometer at the hip, all the time for seven consecutive days, except when showering or sleeping. We used 1-min epoch (60 s) to capture counts of activity. Data points showing >60 min of consecutive zeros of counts were considered as invalid period of use and 10 or more hours per day with valid period was considered to be a valid day. Finally, only participants who had at least five days (one weekend day) of valid accelerometer data were included in this analysis. We use the following classification of sedentary time and physical activity: (a) sedentary time (1–100 counts/min) (e.g. sitting), (b) light-intensity physical activity (101–1952 counts/min) (e.g. walking), (c) moderate-intensity physical activity (1953–5724 counts/min) (e.g. recreational volleyball), (d) vigorous-intensity physical activity (≥5724 counts/min) (e.g. intense cycling) (Freedson et al. 1998).

### Individual characteristics

Sociodemographic variables (sex, age, family SES, employment status, time living in the same household, marital status, number of cars and being a father/mother) were collected using a standardized and pilot-tested questionnaire (Hino et al. 2012). Objective measures of weight and height were used to determine body mass index (BMI). The study protocol was approved by the IRB at the Pontifical Catholic University of Parana (protocol no. 3034/001/1), Brazil and a written informed consent was obtained from each participant.

### Statistical analyses

All study outcomes (physical activity and sedentary time) were examined as continuous variables. Additionally, moderate and vigorous minutes per day of physical activity were combined to determine minutes per day of moderate-to-vigorous physical activity (MVPA) and time spent per day only on bouts of at least 10 min of MVPA. All nine domains of A-NEWS were considered independent variables whereas sex, age, family SES, employment status, time living in the same household, marital status, car ownership and being a father/mother were considered as confounders. Firstly, the sample was described using descriptive statistics. Due to asymmetric distribution physical activity outcomes and sedentary time were transformed using squared roots. Exploratory analyses were conducted through bivariate correlations to identify individual and environmental variables associated with physical activity and sedentary time (*p* < 0.20). Multilevel regressions models were used to account for random effects of census tracts. In the first model, bivariate multilevel regressions analyses were conducted between individual characteristics, environmental features, physical activity and sedentary time, associations showing *p* < 0.20 were included. In the second model, all variables showing significant associations at *p* < 0.20 in the bivariate analyses added to the model. In the third model, all the variables that presented *p* < 0.20 in the bivariate and multivariate analyses (environmental features) were also added into the model. The unstandardized regression coefficients (*B*) and standardized errors (SE) were presented in all models. All analyses were performed in Stata 12.0 using the multi-level mixed regression mode.

## Results

The final and analytical sample comprised 305 participants who provided complete and valid information for all variables. The sample was gender balanced (51.1% of women), and most of participants were employed (80.0%), married or lived with a partner (60.2%) and parents (72.6%). The mean age was 42 years (SD = 12.6) and the mean BMI was 26.7 kg/m^2^ (SD = 4.6). Participants spent, on average, 472.9 min/day in sedentary time, 324.9 min/day in light-intensity activities, 30.5 min/day in MVPA and 11.4 min/day on MVPA with bouts of at least 10 min (Table 1).

**Table 1:** Socio-demographic characteristics in a sample of adults

Variables	Categories	*n* (%) or mean (SD)
Sex, *n* (%)	Female	156 (51.1)
	Male	149 (48.8)
Age [years], average (SD)		42.2 (12.6)
Employment status, *n* (%)	Unemployed	61 (20.0)
	Employed	244 (80.0)
Marital status, *n* (%)	Single, divorced, widower	121 (39.8)
	Married or living with another	183 (60.2)
Car ownership, *n* (%)	No	70 (22.9)
	1 car	138 (45.2)
	2 cars	81 (26.6)
	≥3 cars	16 (5.3)
Being a father/mother, *n* (%)	No	83 (27.4)
	Yes	220 (72.6)
Family SES, *n* (%)	Low	99 (32.7)
	Medium	166 (54.8)
	High	38 (12.5)
Time living in the same household [years], average (SD)		16.1 (12.7)
Time working and studying + commuting (h/week), average (SD)		40.3 (23.9)
BMI measured [kg/m^2^], average (SD)		26.7 (4.6)
Community environment factors, average (SD)	Residential density	282.5 (125.0)
	Land use mix	3.2 (0.6)
	Access to services	3.1 (0.5)
	Street connectivity	2.9 (0.75
	Walking/cycling facilities	2.5 (0.9)
	Aesthetics	2.8 (0.9)
	Pedestrian/traffic safety	2.9 (0.5)
	Crime safety	2.6 (0.4)
	General neighborhood satisfaction	7.0 (2.6)
Sedentary time [min/day], average (SD)		472.9 (112.7)
Light physical activity [min/day], average (SD)		324.9 (100.5)
Moderate-to-vigorous physical activity [min/day], average (SD)		30.5 (24.2)
MVPA bouts 10 of minutes [min/day], average (SD)		11.4 (15.9)

Brazil, 2010 (*n* = 305)

Environment Scale: land use mix = 5 points; general neighborhood satisfaction = 2 points; all other perceptions of the environment = 4 points. The variables, residential density and general neighborhood satisfaction are continuous

*BMI* Body Mass Index, *MVPA* moderate-to-vigorous physical activity, *SD* standard deviation, *SES* socioeconomic status

Table 2 shows the multi-level regression model results for sedentary time as the outcome variable. Significant correlates in the fully adjusted model were family SES [Medium SES (*B* = 35.49), High SES (*B* = 52.84); *p* = 0.009] and residential density (*B* = 0.14; *p* = 0.008) (positive associations), being a father/mother (*B* = −46.36; *p* = 0.009), and street connectivity (*B* = −24.97; *p* = 0.026) (inverse associations). Table 3 presents the equivalent analysis for light-intensity physical activity. In the fully adjusted models, significant correlates were being a father/mother (*B* = 35.71; *p* = 0.025), residential density (*B* = −0.10; *p* = 0.032) and availability of walking and cycling facilities in the neighborhood (*B* = −13.55; *p* = 0.037). Significant correlates of minutes per week of MVPA (Table 4) were sex (*B* = 0.91; *p* < 0.001), neighborhood safety from pedestrian and traffic (*B* = 0.51; *p* = 0.054) (positive associations), BMI (*B* = −0.05; *p* = 0.020) and number of cars [one car (*B* = −1.05; *p* < 0.001), two cars (*B* = −1.14; *p* < 0.001), three cars (*B* = −1.09; *p* = 0.037)] (inverse associations). Finally, number of cars [one car (*B* = −1.18; *p* < 0.001), two cars (*B* = −1.44; *p* < 0.001), three cars (*B* = −1.63; *p* = 0.004)] (inverse association) (data not shown) was correlated with bouts of MVPA.

**Table 2:** Multilevel regression analysis on the contribution of individual and environmental factors in sedentary time

Independent variables	Model 1		Model 2		Model 3	
	*B* (SE)	*p* value	*B* (SE)	*p* value	*B* (SE)	*p* value
Constant					483.93 (43.25)	**<0.001**
Sex (0 = woman; 1 = man)	9.12 (12.40)	0.462	–	–	–	–
Age (years)	−0.97 (0.50)	0.051	–	–	0.32 (0.58)	0.577
BMI measured (kg/m^2^)	−0.63 (1.40)	0.651	–	–	–	–
Family SES (ref = low)						
Medium	46.77 (14.04)	0.001	–	–	35.49 (13.65)	**0.009**
High	66.76 (21.14)	0.002	–	–	52.84 (20.16)	**0.009**
Employment status (0 = no; 1 = yes)	6.33 (15.94)	0.691	–	–	–	–
Time living in the same household (years)	−1.02 (0.50)	0.042	–	–	−0.82 (0.51)	0.107
Marital status (0 = single; 1 = married)	−46.05 (12.78)	<0.001	–	–	−18.33 (14.39)	0.203
Car numbers (ref = no)						
1 car	11.81 (16.05)	0.462	–	–	–	–
2 cars	10.11 (18.28)	0.580	–	–	–	–
3 cars	17.25 (30.85)	0.576	–	–	–	–
Being a father/mother (0 = no; 1 = yes)	−61.39 (14.02)	<0.001	–	–	−46.36 (17.73)	**0.009**
Time working and studying + commuting (h/week)	−0.10 (0.26)	0.718	–	–	–	–
Environmental factors						
Residential density	0.25 (0.05)	<0.001	0.16 (0.05)	0.001	0.14 (0.05)	**0.008**
Land use mix	−7.58 (11.24)	0.500	−4.20 (9.89)	0.671	–	–
Access to services	−11.59 (14.03)	0.409	−3.67 (12.96)	0.777	–	–
Street connectivity	−29.58 (11.54)	0.010	−17.40 (11.22)	0.121	−24.97 (11.22)	**0.026**
Walking/cycling facilities	26.69 (6.92)	<0.001	15.70 (6.87)	0.022	12.20 (8.22)	0.138
Aesthetics	8.19 (7.86)	0.298	9.19 (7.14)	0.198	−3.91 (8.39)	0.641
Pedestrian/traffic safety	−2.59 (13.42)	0.847	−1.45 (12.29)	0.906	–	–
Crime safety	−2.09 (17.98)	0.907	−13.48 (17.37)	0.438	–	–
General neighborhood satisfaction	5.54 (2.51)	0.028	4.98 (2.36)	0.035	2.74 (2.54)	0.281

Brazil, 2010 (*n* = 305 adults; *n* = 32 census tract)

Values shown in bold are significant at *p* ≤ 0.05

*B* unstandardized regression coefficient, *BMI* Body Mass Index, *Min* minutes, *SE* standard error, *SES* socioeconomic status, *Model 1* bivariate analysis, *Model 2* each environmental factor was adjusted for individual variables with *p* ≤ 0.20 (age, family SES, time living in the same household, marital status, being a father/mother), *Model 3* adjusted for individual and environment variables with *p* ≤ 0.20 (age, family SES, time living in the same household, marital status, being a father/mother, residential density, street connectivity, walking/cycling facilities, aesthetics and general neighborhood satisfaction)

**Table 3:** Multilevel regression analysis on the contribution of individual and environmental factors in total light physical activity

Independent variables	Model 1		Model 2		Model 3	
	*B* (SE)	*p* value	*B* (SE)	*p* value	*B* (SE)	*p* value
Constant					331.71 (27.59)	**<0.001**
Sex (0 = woman; 1 = man)	−3.21 (11.04)	0.771	–	–	–	–
Age (years)	1.54 (0.43)	<0.001	–	–	0.40 (0.52)	0.448
BMI measured (kg/m^2^)	0.62 (1.25)	0.621	–	–	–	–
Family SES (ref = low)						
Medium	−16.80 (12.75)	0.188	–	–	−22.15 (14.25)	0.120
High	−37.66 (19.21)	0.050	–	–	−41.62 (23.60)	0.078
Employment status (0 = no; 1 = yes)	10.00 (14.19)	0.481	–	–	–	–
Time living in the same household (years)	1.17 (0.44)	0.008	–	–	0.71 (0.46)	0.127
Marital status (0 = single; 1 = married)	43.16 (11.34)	<0.001	–	–	12.24 (13.29)	0.357
Car numbers (ref = no)						
1 car	20.53 (14.21)	0.148	–	–	14.66 (15.78)	0.353
2 cars	21.46 (16.20)	0.185	–	–	20.52 (19.90)	0.303
3 cars	−8.94 (27.33)	0.743	–	–	13.97 (30.27)	0.644
Being a father/mother (0 = no; 1 = yes)	56.22 (12.38)	<0.001	–	–	35.71 (15.98)	**0.025**
Time working and studying + commuting (h/week)	0.27 (0.23)	0.243	–	–	–	–
Environmental factors						
Residential density	−0.20 (0.48)	<0.001	−0.13 (0.04)	0.003	−0.10 (0.05)	**0.032**
Land use mix	6.14 (10.02)	0.540	5.26 (9.32)	0.572	–	–
Access to services	11.69 (12.50)	0.350	9.32 (11.84)	0.432	–	–
Street connectivity	12.04 (10.38)	0.246	8.19 (10.11)	0.418	–	–
Walking/cycling facilities	−22.94 (6.26)	<0.001	−16.80 (6.17)	0.006	−13.55 (6.50)	**0.037**
Aesthetics	−1.22 (7.10)	0.863	−5.93 (6.58)	0.368	–	–
Pedestrian/traffic safety	0.81 (11.96)	0.946	2.71 (11.27)	0.810	–	–
Crime safety	−4.41 (16.00)	0.783	3.93 (15.70)	0.803	–	–
General neighborhood satisfaction	−2.38 (2.25)	0.290	−2.40 (2.16)	0.267	–	–

Brazil, 2010 (*n* = 305 adults; *n* = 32 census tract)

Values shown in bold are significant at *p* ≤ 0.05

*B* unstandardized regression coefficient, *BMI* Body Mass Index, *LPA* light physical activity, *Min* minutes, *SE* standard error, SES = Socioeconomic Status, *Model 1* bivariate analysis, *Model 2* each environmental factor was adjusted for individual variables with *p* ≤ 0.20 (age, family SES, time living in the same household, marital status, car numbers, being a father/mother), *Model 3* adjusted for individual and environment variables with *p* ≤ 0.20 (age, family SES, time living in the same household, marital status, car numbers, being a father/mother, residential density, street connectivity, aesthetics and general neighborhood satisfaction)

**Table 4:** Multilevel regression analysis on the contribution of individual and environmental factors in measure of moderate-to-vigorous physical activity

Independent variables	Model 1		Model 2		Model 3	
	*B* (SE)	*p* value	*B* (SE)	*p* value	*B* (SE)	*p* value
Constant					4.16 (1.31)	**0.002**
Sex (0 = woman; 1 = man)	−1.05 (0.22)	<0.001	–	–	0.91 (0.24)	**<0.001**
Age (years)	−0.02 (0.01)	0.028	–	–	−0.01 (0.01)	0.475
BMI measured (kg/m^2^)	−0.07 (0.02)	0.005	–	–	−0.05 (0.02)	**0.020**
Family SES (ref = Low)						
Medium	0.08 (0.25)	0.762	–	–	–	–
High	−0.44 (0.38)	0.245	–	–	–	–
Employment status (0 = no; 1 = yes)	1.01 (0.28)	<0.001	–	–	0.66 (0.44)	0.129
Time living in the same household (years)	0.00 (0.01)	0.745	–	–	–	–
Marital status (0 = single; 1 = married)	−0.76 (0.23)	0.001	–	–	−0.35 (0.27)	0.188
Car numbers (ref = no)						
1 car	−1.02 (0.29)	<0.001	–	–	−1.05 (0.29)	**<0.001**
2 cars	−1.18 (0.32)	<0.001	–	–	−1.14 (0.32)	**<0.001**
3 cars	−1.30 (0.54)	0.016	–	–	−1.09 (0.52)	**0.037**
Being a father/mother (0 = no; 1 = yes)	−0.55 (0.26)	0.035	–	–	0.20 (0.32)	0.527
Time working and studying + commuting (h/week)	0.01 (0.00)	0.003	–	–	−0.00 (0.01)	0.663
Environmental factors						
Residential density	0.00 (0.00)	0.536	0.00 (0.00)	0.821	–	–
Land use mix	0.37 (0.19)	0.048	0.09 (0.19)	0.616	–	–
Access to services	0.55 (0.24)	0.026	0.37 (0.23)	0.112	0.28 (0.24)	0.241
Street connectivity	0.20 (0.21)	0.355	0.22 (0.20)	0.277	–	–
Walking/cycling facilities	0.21 (0.13)	0.097	0.16 (0.12)	0.192	0.01 (0.15)	0.947
Aesthetics	0.13 (0.13)	0.340	0.19 (0.13)	0.130	−0.00 (0.16)	0.977
Pedestrian/traffic safety	0.73 (0.23)	0.001	0.60 (0.22)	0.006	0.51 (0.27)	**0.054**
Crime safety	0.82 (0.31)	0.009	0.42 (0.31)	0.176	0.18 (0.35)	0.610
General neighbourhood satisfaction	0.02 (0.04)	0.609	0.00 (0.04)	0.951	–	–

Brazil, 2010 (*n* = 305 adults; *n* = 32 census tract)

Values shown in bold are significant at *p* ≤ 0.05

*B* unstandardized regression coefficient, *BMI* Body Mass Index, *Min* minutes, *MVPA* moderate-to-vigorous physical activity, *SE* standard error, *SES* socioeconomic status, *$* square root (moderate-to-vigorous physical activity (min/day)), *Model 1* bivariate analysis, *Model 2* each environmental factor was adjusted for individual variables with *p* ≤ 0.20 (sex, age, BMI measured, employment status, marital status, car numbers, being a father/mother and time working and studying + commuting), *Model 3* adjusted for individual and environment variables with *p* ≤ 0.20 (sex, age, BMI measured, employment status, marital status, car numbers, being a father/mother, time working and studying + commuting, access to services, walking/cycling facilities, aesthetics, pedestrian/traffic safety and crime safety)

## Discussion

To the best of the authors’ knowledge, this article is the first to evaluate the association between individual characteristics, community environment features and objective-measured sedentary time and physical activity in Brazil, and one of the few conducted in low- and middle- income countries (Jáuregui et al. 2016; Salvo et al. 2016). The results showed that environmental correlates of sedentary time differ from those of light and moderate-to-vigorous intensity physical activity, thus suggesting that these behaviors are somehow independent.

Some associations observed between individual characteristics and sedentary time are consistent with literature. In Belgium (Van Dyck et al. 2010a), living without children was positively associated with sedentary time, and agrees with our findings. Having children at home may lead adults to engage in activities (e.g. childcare and games), which might explain the inverse association with time sedentary time. We have found a positive association between family SES and sedentary time, which is supported by previous study (O’Donoghue et al. 2016). This association is likely to be explained by higher employment and educational status observed in the sample, leading to more time spent working and studying. A positive association between socioeconomic position and minutes per day of sedentary behavior and its interaction with employment status have been reported elsewhere (O’Donoghue et al. 2016). Similar association has been found in a sample of Brazilian adults (Mielke et al. 2014). Nonetheless, further investigation is needed to clarify the role of family SES as sedentary behavior correlate.

Despite the growing evidence on the role of built environment on sedentary behaviors it remains inconsistent and contradictory (O’Donoghue et al. 2016). In our study, residential density was positively, though unexpectedly associated with sedentary time. Van Dyck et al. (2012) found similar results after examining samples from three different countries. This finding may indicate that higher residential density implies that residents have fewer opportunities to engage in more active behaviors (e.g. transport and leisure) hence spending more time at home in engaging in sedentary behaviors (e.g. watching TV). However further examination is required to properly test such connection.

Additionally, street connectivity was also shown to be negatively associated with sedentary behavior. Greater street connectivity increases walking access to and from places hence help buffering or reducing sedentary time spent while driving, which has been found in a recent study (Liao et al. 2016). Additionally, duration and distance can affect the decision on how to commute (e.g. driving versus walking/cycling) (de Hollander et al. 2015). Nevertheless, the accelerometry does not provide domain specific information which prevents further examination of this association.

In our study being father/mother was positively associated with light physical activity. We hypothesized that parents may engage in activities with their children while at home (e.g. playing) and outdoors (e.g. walking children to the school). However, studies that investigating correlates of being father/mother and light intensity physical activity are yet scarce limiting further comparison with our findings. In contrast to expectations, higher density residential and places for walking/cycling were negatively associated with light intensity physical activity. Residential density and walking/cycling facilities have been positively associated with recreational walking (Van Dyck et al. 2013), suggesting that environmental physical activity correlates are domain specific (e.g. leisure or transportation). Furthermore, Van Dyck et al. (2013) have suggested that some level of density is desirable to increase physical activity (e.g. creates more visual interest and increases perceptions of safety), however when residential density is very high, the effect may be the opposite of the expected (Kerr et al. 2016).

Being a woman and BMI were negatively associated with moderate-to-vigorous physical activity. As reported in previous studies it’s expected that men are more engaged in higher intensity leisure physical activities (e.g. sports) than women (Bauman et al. 2012). In addition, individuals with higher BMI are less confident to perform activities at higher intensity (Van Dyck et al. 2010b), which partially explains our findings. Car ownership also showed a negative association with MVPA and MVPA with bouts of 10 min. This result could be explained in part as a consequence of rapid urbanization and access to motorization, especially in countries of low SES (Sallis et al. 2016). Additionally, people who have cars are less likely to commute by walking or cycling (Becerra et al. 2013; Reis et al. 2013). In our study, we defined MVPA through a <1953 counts/min cut-point which includes activities such as slow walking (4.8 km/h). Hence, that could be classified as MVPA what would explain the negative relationship between car ownership and MVPA. Finally, similarly with results from other countries (Gómez et al. 2010), we found that MVPA was associated with neighborhood safety from pedestrian and traffic. This result suggests that people could spent more time at home when feel that it is unsafe doing activities in their neighborhood because of heavy traffic and lack of adequate sidewalks.

This is one of first study investigating the association between individual characteristics of participants, perceived environmental characteristics of the community and physical activity measured objectively in Brazil. To this date, previous studies conducted in Brazil have not examined all items included in the A-NEWS (Hallal et al. 2010; Reis et al. 2013). Hence, our study examined domain specific scores and individual environmental. The analyses included a measure of neighborhood satisfaction, which helps attenuating the neighborhood self-selection effect (McCormack and Shiell 2011). Lastly, the analytical sample did not differ from overall sample, hence response bias was not detected.

However, some limitations should be highlighted. The cross-sectional design of the study prevents establishing causal inference. The sample size may not have been sufficient to identify smaller effect sizes. The use the accelerometer provides a more accurate measure of sedentary time and physical activity than instruments that rely on self-reporting (e.g. questionnaires), though it does not identify the domain in which the activities occurred (e.g. leisure or transportation), hindering the interpretation of the observed associations.

### Conclusion

The findings of this study showed that the individual factors as sex, BMI, family SES, number of cars and being a father/mother were associated with sedentary time and physical activity. Additionally, residential density, street connectivity, walking/cycling facilities and safety from pedestrian and traffic were the environmental correlates associated with sedentary time and physical activity in Brazilian adults. Some associations were in an unexpected direction, showing the need for future studies that seek to better understand this relationship, especially for sedentary time. Hence, studies applying similar measures should be conducted to examine multiple physical activity outcomes allowing comparison with the present study, particularly among populations from low- and middle- income countries. Furthermore, future changes in policies and infrastructure should consider the social context of the community (e.g. family SES) and improvements to promote a safer environment in the neighborhood (e.g. crosswalk, streetlights) to encourage people to be physically active outdoors.
